# Misuse of Aspirin and Associated Factors for the Primary Prevention of Cardiovascular Disease

**DOI:** 10.3389/fcvm.2021.720113

**Published:** 2021-09-03

**Authors:** Yinong Chen, Chun Yin, Qing Li, Luyao Yu, Longyang Zhu, Dayi Hu, Yihong Sun

**Affiliations:** ^1^Peking University Health Science Center, China-Japan Friendship Hospital, Beijing, China; ^2^Department of Cardiology, First Affiliated Hosital of Chongqing Medical University, Chongqing, China; ^3^Heart Center, Peking University, People's Hospital, Beijing, China; ^4^Department of Cardiology, China-Japan Friendship Hospital, Beijing, China

**Keywords:** aspirin, primary prevention, cardiovascular disease, overuse, risk factors

## Abstract

**Background:** The value of aspirin for primary prevention continues to be debated. Data showing whether aspirin use for primary prevention adheres to established guidelines in real world practice are sparse.

**Methods:** A total of 13,104 patients without cardiovascular diseases (CVD) were selected from the DYS-lipidemia International Study of China, a national survey of patients with dyslipidemia in 2012. The CVD risk of the participants were calculated using the 10-year risk of Ischemic Cardiovascular Diseases model. The misuse of aspirin for primary prevention was defined as having CVD risk <10% with daily aspirin. Multivariate logistic regression models were used to explore risk factors associated with aspirin misuse.

**Results:** The proportion of the patients categorized as low, moderate and high risk for CVD were 52.9, 21.6, and 25.4% respectively. The misuse frequency of aspirin was 31.0% (2,147/6,933) in patients with low risk. The misuse of aspirin increased with aging for both men and women. In the multivariate analysis, the independent risk factors associated with aspirin misuse were hypertension, diabetes mellitus, a family history of premature CVD, and elderly age. Level of total cholesterol is negatively associated with aspirin misuse. Patients from low level hospitals are more likely to be taking aspirin inappropriately. Results remained consistent after including 2,837 patients having 10-year risk for CVD between 10 and <20%.

**Conclusion:** The misuse of aspirin for primary prevention is common in patients having CVD risk <10%. There are important opportunities to improve evidence-based aspirin use for the primary prevention of CVD in Chinese patients.

**Clinical Trial Registration:**https://clinicaltrials.gov/, identifier [NCT01732952].

## Introduction

Cardiovascular diseases (CVD) involving atherosclerosis are the leading cause of death both in China and all over the world (1). The evidence for the benefits of aspirin in the secondary prevention of CVD is well-established, but the value of aspirin for primary prevention continues to be debated. The meta-analysis including nine clinical trials showed that aspirin for primary prevention significantly reduced the risk of nonfatal myocardial infarction, but no significant reductions in nonfatal stroke, cardiovascular mortality or all-cause mortality, which is consistent with the former meta-analysis by Antithrombotic Trialists' Collaboration (2, 3). However, the most recent meta-analysis showed that aspirin for primary prevention reduces nonfatal ischemic events while significantly increases nonfatal bleeding events (4). According to the Consensus Document from the ESC Working Group on Thrombosis, aspirin could be considered for the primary prevention of CVD at a risk level of 10-year risk >20% and case-by-case for patients with a risk between 10 and 20% (5). The American guideline did not recommend the use of aspirin in the primary prevention of CVD unless for those with high risk of CVD and no bleeding risk (6). The decision on whether a patient should take aspirin for primary prevention of CVD depends on a balance between CVD and bleeding risks, and this requires the assessment of CVD risk as a first step. For individuals already taking statins that has been proven to be effective for primary prevention of CVD, the absolute risk is lower compared to those without statins and the need for aspirin remains unclear (7).

Data showing whether aspirin use for primary prevention adheres to established guidelines in real world practice are sparse. A large U.S. nationwide registry from 2008 to 2013 showed that more than 1 in 10 patients were receiving inappropriate aspirin therapy for primary prevention (8). Understanding the associated risk factors for aspirin misuse is important for optimizing the treatment for the primary prevention (9). In the present analysis, we described the contemporary patterns of aspirin use for primary prevention for CVD in Chinese patients taking lipid-lowering drugs and explored the risk factors associated with aspirin misuse.

## Methods

### Study Sample

This study was a *post-hoc* analysis of a multi-center cross-sectional study, the DYS-lipidemia International Study of China (the DYSIS-China), carried out at 122 centers between April 2012 and October 2012. The study design of the DYSIS-China has been published previously (10). Briefly, outpatients were consecutively enrolled if they were: 45 years or older and had been taking any lipid-lowering drugs for at least 3 months. In this analysis, we excluded the participants with a history of any CVD, including coronary heart disease, peripheral arterial disease, and stroke. The study was registered at clinicaltrials.gov under NCT01732952. All patients provided informed consent before beginning the study, and the study protocol was approved by the Ethics Committee of each clinic center.

### Data Collection and Definitions

The data for demographic characteristics, co-morbid diseases, and medication were recorded, as was information related to the treating physicians (cardiologists, endocrinologists, geriatricians, internists, and neurologists) and corresponding hospitals. CVD risk was evaluated based on the 10-year risk of Ischemic Cardiovascular Diseases (ICVD) model, which includes sex, age, systolic blood pressure, diabetes mellitus, total cholesterol, smoking, and body mass index (11). Participants were divided into three categories on the basis of CVD risk: low risk if the 10-year CVD risk was <10%, moderate risk if the 10-year CVD risk was between 10 and 20%, and high risk if the 10-year CVD risk was >20%. Misuse of aspirin for primary prevention was defined as having <10% cardiovascular disease risk with daily aspirin use.

### Statistical Analysis

Continuous variables were expressed as mean ± standard deviation (SD) if they tended to be normal and compared using one-way analysis of variance (ANOVA). Categorical variables were summarized using number and percentages and compared using the Pearson chi-squared test or Fisher exact test. Multivariable logistic regression was to identify factors associated with misuse of aspirin in low risk patients. Covariates were selected according to baseline differences between aspirin and non-aspirin groups. The results were expressed as odds ratios (ORs) and 95% confidence intervals (CIs). The misuse of aspirin was defined as the treatment of a patient with a CVD risk <10% with aspirin. The ESC Consensus document suggests that aspirin use in patients with a risk between 10 and 20% should be engaged in a case-by-case discussion (5). Given this recommendation, we performed sensitive analyses by including patients with a risk <20% as the misuse cohort. The frequency of aspirin misuse was then re-evaluated. A statistically significant difference was considered at the two-tailed level of *p* <0.05. Analyses were conducted from June to September 2020. Statistical analyses were performed using SAS version 9.4 (SAS Institute Inc., Cary, NC, USA).

## Results

### Patient Characteristics

**Figure 1:**
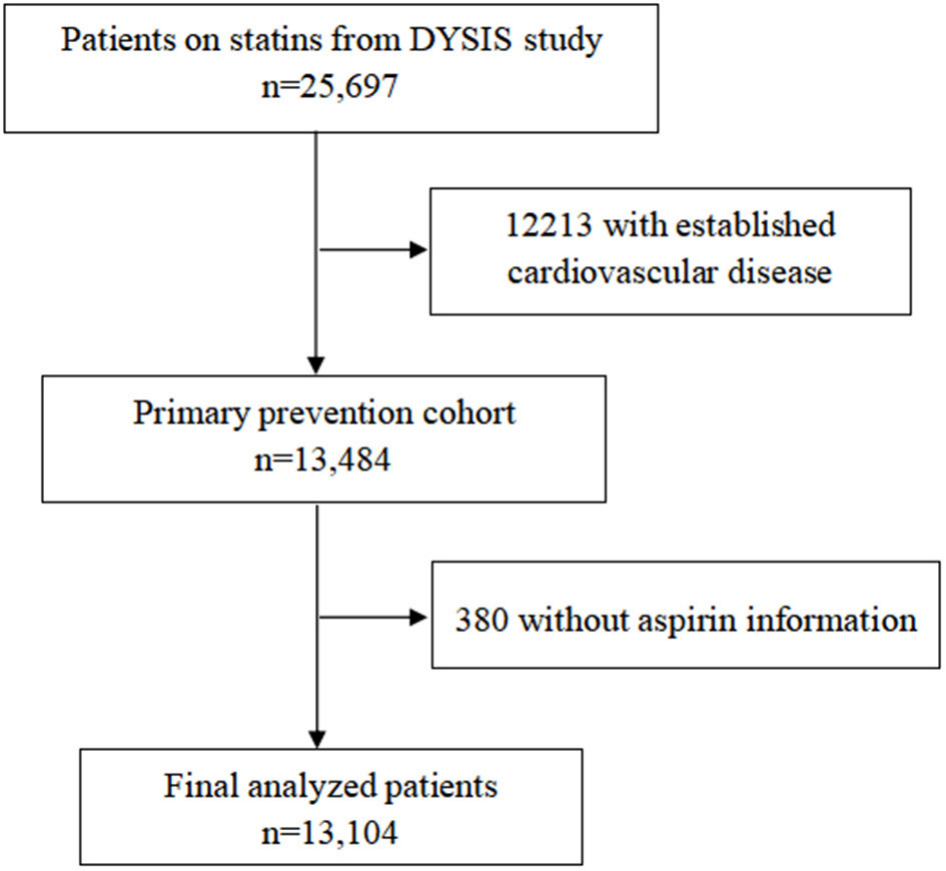
Flow chart of study participants.

A total of 13,104 individuals were analyzed (Figure 1) with an average age of 63.2 ± 10.3 years. 55.0% (7,204) were female. Overall, 52.9% (6,933/13,104), 21.7% (2,837/13,104), and 25.4% (3,334/13,104) of participants were categorized as low, moderate, and high CVD risk, respectively. 53.7% of the patients were taking anti-hypertensive medication and 34.4% were taking anti-diabetic medication. The average total cholesterol level and low-density lipoprotein cholesterol level were 189.4 ± 46.4 mg/dL and 108.2 ± 38.7mg/dL, respectively. The characteristics of the study population on the base of 10 years risk for CVD are shown in Table 1.

**Table 1:** Characteristics based on 10-year risk for CVD.

**Variables**	**10-year risk for CVD**			***p-*value**
	** <10*%* (*n* = 6,933)**	**10–20% (*n* = 2,837)**	**>20% (*n* = 3,334)**	
Age, years	57.7 ± 7.9	66.4 ± 8.5	71.8 ± 8.8	<0.001
≥65 years	1,276 (18.4)	1,657 (58.4)	2,677 (80.3)	<0.001
Female	2,827 (55.2)	1,637 (57.7)	1,740 (52.2)	0.001
Current smoker	742 (10.7)	360 (12.7)	470 (14.1)	<0.001
Sedentary lifestyle	1,102 (15.9)	491 (17.3)	677 (20.3)	<0.001
BMI, kg/m^2^	24.3 ± 3.2	24.9 ± 3.3	25.4 ± 3.3	<0.001
Waist circumference, cm	85.7 ± 11.0	86.7 ± 10.7	88.0 ± 11.0	<0.001
Diabetes	1,927 (27.8)	1,115 (39.3)	1,770 (53.1)	<0.001
Hypertension	2,690 (38.8)	1,955 (68.9)	2,991 (89.7)	<0.001
Family history of premature CVD	506 (7.3)	238 (8.4)	237 (7.1)	0.151
SBP, mmHg	123.1 ± 11.4	132.0 ± 12.2	143.1 ± 15.4	<0.001
DBP, mmHg	76.8 ± 8.5	78.9 ± 9.5	81.2 ± 10.7	<0.001
Uric acid, μmol/L	313.6 ± 92.7	323.9 ± 95.8	335.3 ± 99.9	<0.001
TC, mg/dL	189.4 ± 46.4	185.6 ± 46.4	181.7 ± 46.4	<0.001
LDL-C, mg/dL	108.2 ± 38.7	104.4 ± 38.7	104.4 ± 38.7	<0.001
HDL-C, mg/dL	50.3 ± 15.5	50.3 ± 15.5	50.3 ± 15.5	<0.001
TG, mg/dL	150.6 (97.5, 212.64)	141.8 (106.3, 203.8)	141.8 (106.3, 203.8)	0.043
Non-HDL-C, mg/dL	139.2 ± 46.4	135.3 ± 42.5	131.4 ± 42.5	<0.001
Anti-hypertensive medications	1,692 (24.4)	1,390 (49)	2,200 (66)	<0.001
Anti-diabetic medications	1,511 (21.8)	1,245 (43.9)	150 (45)	0.038
Hospital level				0.162
Tertiary	3,217 (46.4)	1,260 (44.4)	1,520 (45.6)	
Secondary	1,615 (23.3)	667 (23.5)	823 (24.7)	
Primary	2,101 (30.3)	908 (32.0)	990 (29.7)	

*Data were expressed as mean ± SD, n (%) or median (inter-quartile range)*.

*CVD, cardiovascular disease; BMI, body mass index; SBP, systolic blood pressure; DBP, diastolic blood pressure; TC, total cholesterol; TG, triglycerides; LDL-C, low-density lipoprotein cholesterol; HDL-C, high-density lipoprotein cholesterol*.

### Aspirin Uses and Dosage

The overall prevalence of aspirin use was 38.6% (5,056/13,104), with 40.8% (2,407/5,900) in men and 36.8% (2,649/7,204) in women. The majority of patients taking aspirin (95.0%) received a dose from 75 mg to 100 mg/d, with only 4.1% taking a dose <75 mg/d and 0.9% taking a dose >100 mg/d. The rate of aspirin use also increased with increasing 10-year CVD risk, with rates of 31.0% (2,147/6,933), 42.9% (1,218/2,837), and 50.7% (1,691/3,334) in the low, moderate, and high CVD risk groups, respectively (Supplementary Figure 1). The rate of aspirin misuse was 16.4% (2,147/13,104) in the overall cohort.

### Aspirin Misuse in Patients With Low Risk for CVD: Univariable and Multivariable Analysis

Among the patients with low risk, 31.0% (2,147/6,933) were receiving aspirin inappropriately. Baseline characteristics comparison between those with and without aspirin in low risk group are summarized in Table 2. Patients with aspirin were more likely to be male, elderly, a current or former smoker, and more likely to have hypertension, diabetes, and family history of premature CVD. The frequency of aspirin misuse increased from 18.5% among participants aged <50 years to a peak value of 37.5% among participants aged >70 years. Patients receiving aspirin inappropriately had lower level of total cholesterol, LDL-C and TG. Patients with aspirin misuse were more likely to be prescribed antihypertensive medications (*p* <0.001). The rate of aspirin misuse was higher among those treated in low-level hospitals (Table 2).

**Table 2:** Comparison between patients with and without aspirin in low risk group.

**Variables**	**Aspirin (*n* = 2,147)**	**Non-aspirin (*n* = 4,786)**	***p-*value**
Age, years	58.3 ± 7.7	57.5 ± 7.9	<0.001
Female	1,117 (52.0)	2,707 (56.6)	<0.001
Current or former smoker	541 (5.2)	1,060 (22.2)	0.017
BMI, kg/m^2^	24.5 ± 3.2	24.3 ± 3.2	0.064
Diabetes	653 (30.4)	1,274 (26.6)	0.001
Hypertension	1,198 (55.8)	1,492 (31.2)	<0.001
Family history of premature CV	229 (10.7)	277 (5.8)	<0.001
SBP, mmHg	123.4 ± 11.6	123.0 ± 11.3	0.284
DBP, mmHg	76.9 ± 8.3	76.7 ± 8.6	0.182
TC, mg/dL	181.7 ± 46.4	193.3 ± 46.4	<0.001
LDL-C, mg/dL	104.4 ± 38.7	112.1 ± 38.7	<0.001
HDL-C, mg/dL	50.3 ± 15.5	50.3 ± 15.5	0.008
TG, mg/dL	177.2 ± 132.9	186.1 ± 150.6	<0.001
Non-HDL-C, mg/dL	131.4 ± 42.5	143.0 ± 42.5	<0.001
Anti-hypertensive therapy	1,094 (51.0)	1,279 (26.7)	<0.001
Anti-diabetic therapy	610 (28.4)	1,186 (24.8)	0.790
Hospital level			0.016
Tertiary	985 (45.9)	2,233 (46.7)	
Secondary	544 (25.3)	1,070 (22.4)	
Primary	618 (28.8)	1,483 (31.0)	

*Data were expressed as mean ± SD or n (%)*.

*BMI, body mass index; CVD, cardiovascular disease; SBP, systolic blood pressure; DBP, diastolic blood pressure; TC, total cholesterol; TG, triglycerides; LDL-C, low-density lipoprotein cholesterol; HDL-C, high-density lipoprotein cholesterol*.

Multivariate logistic regression analysis showed that hypertension (OR = 2.69), diabetes (OR=1.80), family history of premature CVD (OR = 1.60) were significantly associated with aspirin misuse (Table 3). Every 5 years increase in age was associated with a 2.2% higher rate of aspirin misuse (OR = 1.02). Higher level of total cholesterol was inversely associated with aspirin misuse (OR = 0.86). In addition, patients treated in low-level hospitals had a higher risk of aspirin misuse than those treated in tertiary hospitals (OR = 1.38) (Table 3). The comparison of patients with and without aspirin in patients having <20% risk for CVD is showed in Supplementary Table 1. The results of multivariable logistic analysis remained consistent after including all patients having <20% risk for CVD (Supplementary Table 2).

**Table 3:** Independent risk factors of aspirin overuse by multivariate logistic regression analysis for patients with low risk.

**Variables**	**OR (95*%*CI)**	***p-*value**
Age (per 5 years)	1.02 (1.01, 1.03)	<0.001
Family history of premature CVD	1.60 (1.31, 1.94)	<0.001
Hypertension	2.69 (2.40, 3.00)	<0.001
Diabetes	1.80 (1.58, 2.05)	<0.001
Total cholesterol, mg/dL	0.86 (0.82, 0.91)	<0.001
Low-level hospital (vs. tertiary)	1.38 (1.22, 1.55)	<0.001

*The multivariate logistic regression model included the following factors: age, male, current or former smoker, Family history of premature CVD, diabetes, hypertension, BMI, total cholesterol, LDL-C, HDL-C, TG, Non-HDL, anti-hypertensive therapy, hospital level by using forward stepwise*.

*OR, odds ratios; CVD, cardiovascular disease; BMI, body mass index; LDL-C, low-density lipoprotein cholesterol; HDL-C, high-density lipoprotein cholesterol; TG, triglycerides*.

## Discussion

In this cohort of Chinese patients without a history of CVD, 52.9% of all the patients were at low risk of CVD on the basis of the 10-year CVD risk calculator. The overall rate of aspirin misuse for the primary prevention of CVD was 16.4 % in the overall cohort. Almost one third of the patients having low risk for CVD were taking aspirin. The patients having low risk for CVD are more likely to be taking aspirin if they had family history of CVD, hypertension, or diabetes. Lower level of total cholesterol is associated with higher risk of aspirin misuse. Patients from low-level hospitals were more likely to be taking aspirin for primary prevention of CVD. Our results provide important information for aspirin misuse for primary CVD prevention in contemporary practice.

In this study, about one third of the patients having <10% of 10 year risk for CVD were taking aspirin inappropriately. This accounts for 39.7% of all the aspirin users. One study of U.S cohort showed 10% of the participants used aspirin inappropriately defined as aspirin therapy in patients with aspirin use along with a 10- year risk of a CVD event <6% (8). In a nationwide integrated health system of US Veterans, many patients are prescribed low-dose aspirin outside of the guidelines. Besides, a considerable number of patients age >70 years take aspirin outside the ACC/AHA recommendation (12). We also found the frequency of aspirin misuse increased with aging. The Multi-Ethnic Study of Atherosclerosis showed that the prevalence of aspirin use increased with aging regardless of the CVD risk of the patients (13). The National Health and Nutrition Examination Surveys showed that about 45.3% of adults aged ≥ 75 years took low dose aspirin daily for primary prevention. In current guidelines, aspirin was contraindicated in patients aged >70 years for the primary prevention of CVD (5, 6). Our findings indicated that an evidence based decision making strategy for primary prevention of elderly patients should be employed in real world practice. Patients aged > 60 years have a 2-fold greater risk of all bleeding events rate (31.96 vs. 16.48%) and a 4-fold greater risk of severe gastrointestinal bleeding (12.83 vs. 2.96%) as compared with younger patients (14). A population-based cohort study found that the risk of non-major bleeding was unrelated to age, but risk of major bleeding was 3 times higher for those age ≥75 years, particularly for fatal bleeding (15). The results of one meta-analysis indicated the absolute incidence of hospitalization for major bleeding increased with each decade of older age, from 2.48 events per 1,000 person-years in those ages 50–59 years to 10.60 events per 1,000 person-years in those age 80 years or older (16). However, a retrospective cohort study used data from the Korean National Health Insurance Service showed that aspirin lowered the risks of primary major cardiovascular events and cancer without increasing the bleeding risk in Koreans with CV risks aged between 60 and 80 years. But only 10 percent of these patients were taking lipid lowering drugs (17). Prophylactic aspirin in primary prevention of CVD is potentially harmful and should not be used in patients aged >70 years.

Our study found that patients with hypertension but low risk of CVD were more likely to receive aspirin treatment inappropriately. A Chinese survey found that about 30% of hypertensive patients were taking aspirin for primary prevention, of which 44.5% having low risk (18). The high rate of aspirin use in patients with hypertension could be partly explained by the definition of the 10 year risk calculators in the Chinese guideline, in which hypertension alone equals to three other risk factors. We also found that patients taking anti-hypertensive medication or anti-diabetic medication was more likely to use aspirin inappropriately. This may be due to the fact that patients with hypertension or diabetics have better health consciousness than those without treatment. The proportion of smokers were less in low risk patients with aspirin misuse than those without (19). The risk assessment model is warranted to be optimized as the control of hypertension has been improving and prevalence of statins treatment for primary prevention is increasing.

The optimal use of aspirin is controversial in patients with diabetes, although diabetes contributes a very high risk for CVD. The prevalence of aspirin use in diabetes is higher in our study compared to that reported in a cohort of diabetes patients (20). This recognized that diabetes itself is a high risk factor in the assessment of CVD risk for primary prevention (5, 19). However, randomized controlled trials have not consistently shown significant a reduction in CVD events with aspirin compared with placebo (21, 22). Recently, the ASCEND trial provided the contemporary evidence for the aspirin use in patients with diabetes. The lack of significant benefit of aspirin observed using the original prespecified primary endpoint and the small magnitude of clinical effect (1.1% absolute risk reduction) indicate that aspirin does not have a definitive indication in primary prevention for patients with diabetes. The results of ASCNED trial also indicated that 40% of the patients with diabetes, mostly taking statins, had 10 year risk of CVD <10% (23). The meta-analysis by USPSTF showed that patients with diabetes had a 55% risk of higher extra-cranial bleeding, a 74% higher risk of hemorrhagic stroke, and 36% higher risk of hospitalization for major bleeding events compared with non-diabetic patients (16). Consistent with these previous studies, our analysis revealed that patients with diabetes were also at higher risk of aspirin misuse, which would put these patients at higher risk of major bleeding. Thus, the use of aspirin for primary prevention of CVD in patients with diabetes should be based on the individualized assessment of benefit and risk (7). The mere presence of diabetes is apparently not enough for aspirin to confer a benefit that clearly outweighs the risk of bleeding.

Align with previous study, our results indicate there was significant practice-level variation in the misuse of aspirin (8). There was a moderate but significantly difference in the aspirin misuse in low-level hospitals compared to tertiary level hospitals in this study. It is reported that the extent of practice-level variation using the median rate ratio was 1.63, which suggests that between 2 “identical” patients treated at 2 randomly chosen practices, patient was 63% more likely to receive aspirin inappropriately than another patient with similar characteristics because of the practice where they were receiving care. The regional variation was also noted in aspirin misuse (8). These analyses suggest that there is much work to be done to educate practitioners and practices about the need to ascertain risk by using well-validated methods before initiating medications, such as aspirin, for primary CVD prevention.

### Strengths and Limitations

Our study was a large cross-sectional study of aspirin use for primary prevention of CVD among patients treated with anti-dyslipidemia medication. The use of aspirin for primary prevention of CVD was challenged in part because of the well-established efficacy and safety of statins for this purpose. A study of Korean patients with hypertension showed that aspirin alone (HR = 0.62), statins alone (HR = 0.48) were independently associated with reductions in all-cause mortality, while the addition of aspirin to statins was not associated with an additive benefit in reducing total mortality or cardiovascular mortality (24). We calculated the 10-year risk of CVD using the cholesterol level on statin treatment. So, this provided the opportunity to investigate the possibility of aspirin use on top of statins for the primary prevention of CVD. However, several limitations should be mentioned. First, this is a *post-hoc* analysis of the DYSIS China study, which is a cross-sectional survey of the use of statins in patients with dyslipidemia. Compared to the guidelines from American and Europe, Chinese guidelines did not update the recommendation of the aspirin use in primary prevention until recently, partly because there was no Chinese recruited in the recent trials, for example ASCEND, ARRIVE, and ASPREE trial. Besides, the control of major risk factors, such as hypertension and diabetes, is far beyond ideal in the Chinese population compared to the western population (25). The use of aspirin remained as the choice of primary prevention for CVD. This study provided important information for improving the optimal use of aspirin for clinicians, albeit the data were collected in 2012. Second, the patients of this cohort were taking statins for dyslipidemia and might reflect a strictly pattern of drug use in China and that conclusions are not therefore generalizable to the general population. Third, aspirin use for primary prevention was self-reported. Because aspirin is available over the counter with no need for prescriptions, aspirin use could be underreported, and the rates of inappropriate use in patients with 10-year risk for CVD <10% could be higher. Forth, all the patients were taking statins, and 53.8% had received anti-hypertensive treatments, leading to underestimation of the CVD risk of the cohort. A sensitivity analysis for the independent risk factors in patients having <20% risk for CVD yielded results similar to those for the low risk group. Fifth, we did not assess the 10 year risk for CVD based on Framingham risk and SCORE because ICVD has been derived and validated in Chinese population. Both ICVD risk and Framingham risk were used to predict the 10 year risk for ischemic CV events. However, the analysis of two large Chinese cohorts indicated that Framingham models significantly overestimated the coronary heart disease risk (25, 26). The SCORE risk is used to predict CV death and has not been validated in Chinese population. Finally, we did not assess bleeding risk because the relevant information was not collected in DYSIS-China survey. Therefore, aspirin might be used inappropriate for some of the patients with a moderate to high risk of CVD with or without high bleeding risk. Despite these limitations, our study provides an overview of aspirin use for primary prevention of CVD in dyslipidemia patients receiving statins and reveals potential approaches to improving aspirin utilization for CVD prevention in China.

## Conclusion

Aspirin misuse was common in patients with 10-year risk of CVD <10% for the primary prevention in China. Given current trial evidence, aspirin should no longer be recommended for all primary prevention patients, and now probably only for a minority of them. After optimal control of cardiovascular risk factors, involving in most patients properly titrated statin therapy and blood pressure control, clinicians should assess cardiovascular risk as the first step to avoid the harm of aspirin in low risk patients.

## Data Availability Statement

The raw data supporting the conclusions of this article will be made available by the authors, without undue reservation.

## Ethics Statement

The studies involving human participants were reviewed and approved by the Ethics Committee of each clinic center. The patients/participants provided their written informed consent to participate in this study.

## Author Contributions

YS was the corresponding author and contributed to the draft review and revision. Material preparation and data collection were performed by YC, CY, QL, and LY. Data analysis was performed by LZ and DH. The first draft of the manuscript was written by YC and CY. All authors commented on previous versions of the manuscript, read and approved the final manuscript, and contributed to the study conception and design.

## Conflict of Interest

This study received funding from a research grant from MoShaDong China. The funder was not involved in the study design, collection, analysis, interpretation of data, the writing of this article or the decision to submit it for publication. All authors declare no other competing interests. The authors declare that the research was conducted in the absence of any commercial or financial relationships that could be construed as a potential conflict of interest.

## Publisher's Note

All claims expressed in this article are solely those of the authors and do not necessarily represent those of their affiliated organizations, or those of the publisher, the editors and the reviewers. Any product that may be evaluated in this article, or claim that may be made by its manufacturer, is not guaranteed or endorsed by the publisher.
