# Supplementary figures and images for: Misuse of Aspirin and Associated Factors for the Primary Prevention of Cardiovascular Disease
Source: Front Cardiovasc Med. 2021 Sep 3;8:720113. doi: 10.3389/fcvm.2021.720113 (PMC8446611; doi:10.3389/fcvm.2021.720113)

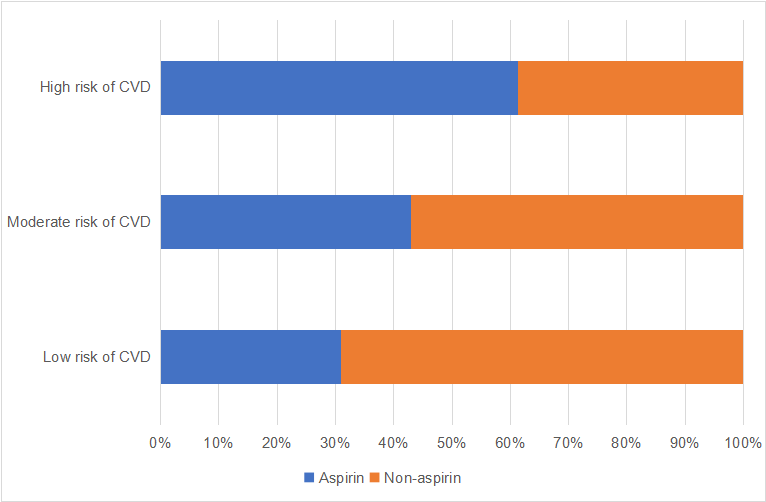

Supplement: Supplementary file 1 [file Image_1.TIF]
