# Supplementary material for: Misuse of Aspirin and Associated Factors for the Primary Prevention of Cardiovascular Disease
Source: Front Cardiovasc Med. 2021 Sep 3;8:720113. doi: 10.3389/fcvm.2021.720113 (PMC8446611; doi:10.3389/fcvm.2021.720113)
Supplement: Supplementary file 2 [file Data_Sheet_1.docx]

**Supplementary tables**

**Supplementary Table 1.** Comparison of patients with and without aspirin in patients having 10-year risk for CVD <20% (*n*=9770)

| **Variables** | **Aspirin (*n*=3365)** | **Non-aspirin (*n*=6405)** | ***p-*value** |
| --- | --- | --- | --- |
| Age, years | 62.3±8.1 | 61.5±9.4 | 0.270 |
| Female | 1831(54.4) | 3633 (56.7) | 0.060 |
| Current or former smoker | 667 (19.8) | 1729 (27.2） | 0.021 |
| BMI, kg/m^2^ | 24.6±3.2 | 24.7±3.3 | 0.495 |
| Diabetes | 1211 (36.4) | 1831 (28.5) | 0.007 |
| Hypertension | 2254 (67.0) | 2391 (37.3) | <0.001 |
| Family history of premature CVD | 286 (8.6) | 458(7.1) | 0.120 |
| SBP, mmHg | 128.4±14.6 | 130.0±15.9 | 0.007 |
| DBP, mmHg | 78.9±9.3 | 78.7±9.6 | 0.291 |
| TC, mg/dL | 174.0±42.5 | 189.4±46.4 | <0.001 |
| LDL-C, mg/dL | 100.5±35.8 | 108.2±38.7 | <0.001 |
| HDL-C, mg/dL | 50.3±11.6 | 50.3±15.5 | 0.001 |
| TG, mg/dL | 177.2±132.9 | 177.2±150.6 | 0.261 |
| Non-HDL-C, mg/dL | 131.4±46.4 | 139.2±42.5 | <0.001 |
| Anti-hypertension therapy | 1891 (56.1) | 2306 (36.0) | <0.001 |
| Anti-diabetic therapy | 1210 (36.0) | 1681 (26.2) | 0.005 |
| Hospital level |  |  | <0.001 |
| Tertiary | 1588 (43.9) | 2889 (45.1) |  |
| Secondary | 879 (26.1) | 1403 (21.9) |  |
| Primary | 896 (29.8) | 2113 (33.0) |  |

*CVD, cardiovascular disease; BMI, body mass index;* *SBP, systolic blood pressure; DBP, diastolic blood pressure; TC, total cholesterol; LDL-C, low-density lipoprotein cholesterol; HDL-C, high-density lipoprotein cholesterol;* *TG, triglycerides.*

**Supplementary Table 2.** Independent risk factors of aspirin misuse by multivariate logistic regression analysis for patients having 10-year risk <20%

| **Variables** | **OR (95*%*CI)** | ***p-*value** |
| --- | --- | --- |
| Age (per 5 years) | 1.04(1.02, 1.07) | <0.001 |
| Family history of premature CVD | 1.23(1.03, 1.48) | <0.001 |
| Hypertension | 2.33(2.02, 2.67) | 0.002 |
| Diabetes | 1.54(1.38, 1.73) | <0.002 |
| Total cholesterol, mg/dL | 0.81(0.77, 0.85) | <0.001 |
| Low-level hospital (vs tertiary) | 1.21(1.10, 1.45) | <0.001 |

*OR, odds ratios; CVD, cardiovascular disease.*
